# Escores Prognósticos de Mortalidade na Cirurgia Cardíaca para Endocardite Infecciosa

**DOI:** 10.36660/abc.20200070

**Published:** 2020-04-06

**Authors:** Alexandre Bahia Barreiras Martins, Cristiane da Cruz Lamas

**Affiliations:** 1 Unidade Cardiointensiva Clínica São Vicente da Gávea Rio de JaneiroRJ Brasil Unidade Cardiointensiva - Clínica São Vicente da Gávea, Rio de Janeiro, RJ - Brasil; 2 Coordenação de Ensino e Pesquisa Instituto Nacional de Cardiologia Rio de JaneiroRJ Brasil Coordenação de Ensino e Pesquisa - Instituto Nacional de Cardiologia, Rio de Janeiro, RJ - Brasil; 3 Escola de Ciências da Saúde Universidade do Grande Rio Rio de JaneiroRJ Brasil Escola de Ciências da Saúde - Universidade do Grande Rio (Unigranrio), Rio de Janeiro, RJ - Brasil; 4 Centro Hospitalar Instituto Nacional de Infectologia Evandro Chagas Fiocruz Rio de JaneiroRJ Brasil Centro Hospitalar - Instituto Nacional de Infectologia Evandro Chagas - Fiocruz, Rio de Janeiro, RJ – Brasil

**Keywords:** Endocardite/cirurgia, Mortalidade Hospitalar, Cirurgia Cardíaca/mortalidade, Prognóstico, Indicadores

O artigo de Pivatto F Jr et al.,^1^ nos permite discutir a importante questão dos escores prognósticos em pacientes submetidos à cirurgia cardíaca para endocardite infecciosa (EI).^1^ O manejo da EI do lado esquerdo geralmente envolve cirurgia durante a admissão índice, e o principal desafio é identificar rápida e corretamente pacientes de alto risco e transferi-los para instituições com uma equipe cirúrgica com experiência em cirurgia de endocardite.

Os escores prognósticos são importantes por várias razões: uma estimativa razoável do risco de morte é importante na tomada de decisão clínica em relação à indicação cirúrgica; a estimativa é necessária para informar os pacientes e suas famílias sobre o risco cirúrgico; a estratificação de risco permite uma comparação razoável dos resultados da cirurgia cardíaca, para que cirurgiões e hospitais que tratam pacientes de alto risco não pareçam ter piores resultados do que outros.^2^ Para que a mortalidade operatória permaneça uma medida válida da qualidade da assistência, ela deve estar relacionada ao perfil de risco dos pacientes submetidos à cirurgia.^2^

O Euroscore I, publicado em 1999, avaliou 19.030 pacientes submetidos à cirurgia cardíaca em 8 países da Europa, estudando 97 fatores de risco para óbito, dentre os quais foram selecionados aqueles que significativamente afetaram o prognóstico cirúrgico.^2^ Essas variáveis são apresentadas na Tabela 1 . Neste estudo, apenas 30% foram submetidos à cirurgia valvar e o número de indivíduos que apresentou endocardite não é mencionado.^2^

**Tabela 1:** – Variáveis incluídas em escores prognósticos de cirurgia cardíaca (Euroscore I e II e STS-IE)

Variáveis	EuroScore I	EuroScore II	STS-IE Score
Idade			
Gênero			
Peso			
Altura			
Índice de Massa Corpórea			
Diabetes Mellitus			
Doença Pulmonar Crônica			
Arteriopatia Extracardíaca			
Doença Arterial Periférica			
Disfunção Neurológica			
Baixa Mobilidade			
Cirurgia cardíaca prévia			
Número de cirurgias prévias			
Cirurgia valvar prévia			
Insuficiência renal em tratamento conservador			
Insuficiência Renal em Hemodiálise			
Creatinina Sérica/ Clearance de Creatinina			
Arritmia			
Hipertensão Arterial Sistêmica			
***Endocardite infecciosa em atividade***			
Terapia imunossupressora			
Infarto do miocárdio recente			
Choque cardiogênico			
Uso de inotrópicos			
Balão intraaórtico			
Classificação NYHA (New York Heart Association)			
Cirurgia não coronariana			
Angina Instável (CCS IV)			
Estado Crítico Pré-operatório			
Fração de Ejeção do Ventrículo Esquerdo			
Hipertensão Arterial Pulmonar			
Ressuscitação			
Procedimento de urgência			
Peso da Intervenção:			
Procedimento único não coronariano			
2 Procedimentos			
3 Procedimentos			
Ruptura septal pós Infarto do miocárdio			

O Euroscore II, publicado em 2012,^3^ teve como objetivo atualizar o primeiro modelo, avaliando 22.381 pacientes de 43 países do mundo, incluindo locais fora da Europa, para criar um escore mais confiável, incorporando novas variáveis e ajustando outras ( Tabela 1 ). Nesta época, já se sabia que o Euroscore2 superestimava o risco cirúrgico, pois progresso técnico em cirurgia cardíaca havia sido alcançado ao longo da década anterior, com uma diminuição da mortalidade ajustada pelo risco. As melhorias no Euroscore foram: clearance de creatinina como uma medida melhor da função renal do que os valores séricos de creatinina; a angina instável definida pelo uso de nitratos intravenosos estava desatualizada; o peso da intervenção não foi avaliado adequadamente no modelo anterior (por exemplo, a troca valvar aórtica com ou sem cirurgia de revascularização do miocárdio concomitante tinha o mesmo peso) e algumas variáveis contínuas, como número de cirurgias cardíacas anteriores e pressões sistólicas da artéria pulmonar, foram tratadas como dicotômicas.^3^

A curva ROC ( *Receiver Operating Characteristic* ) dos escores mostrou uma área abaixo da curva (AUC, *area under the curve* ) de 0,78 para o Euroscore logístico e aditivo e de 0,80 para o Euroscore II. Uma crítica ao modelo é que, embora países não europeus tenham sido incluídos, a grande maioria dos pacientes era da Espanha, França, Itália e Reino Unido, que contribuíram com 19, 16, 15 e 12 centros, respectivamente.^3^Quanto à América Latina, o Brasil contribuiu com dados de 4 centros, Argentina 1 e Uruguai 1. Além disso, o modelo não analisou separadamente a cirurgia valvar. De fato, apenas 2,2% dos pacientes (497 em números absolutos) com EI ativa foram incluídos.^4^ Uma limitação descrita no estudo foi que todos os centros participaram voluntariamente, o que introduz um viés de seleção para os dados.^3^

Pacientes com EI precisam ser avaliados minuciosamente. Se tomarmos o perfil habitual de um paciente com EI operado no Instituto Nacional de Cardiologia, por exemplo, ele terá uma creatinina sérica acima do normal, com escore de 2 pontos; doença ativa (em tratamento com antibiótico para EI no momento da cirurgia), com escore de 3 pontos e disfunção ventricular esquerda pelo menos moderada, com escore de 1 ponto, ou seja, o Euroscore dele seria de 6 pontos e a mortalidade prevista seria superior a 11%. Não raramente, esse paciente foi submetido a cirurgia cardíaca anterior (já que mais de um terço tem valvopatia reumática e cerca de 10% tiveram EI anterior), o que acrescenta 3 pontos ao escore.^4 - 6^Portanto, o Euroscore I não discrimina bem esse subgrupo de pacientes, pois a maioria provavelmente irá apresentar um escore de 6 ou mais. Patrat-Delon et al.,^7^ estudando 149 pacientes operados devido a EI na França, entre 2002 e 2013, cuja mortalidade hospitalar foi de 21%, chegaram a uma conclusão semelhante em relação ao EuroSCORE II: subestimou a mortalidade em pacientes com mortalidade prevista acima de 10%.^7^

O escore da *Society of Thoracic Surgeons–Infective Endocarditis* (STS-IE), publicado em 2011,^8^ tem suas variáveis mostradas esquematicamente na Tabela 1 . No subgrupo de pacientes norte-americanos com EI estudados, dos 13.617 pacientes, apenas pouco mais da metade apresentava endocardite ativa no momento da cirurgia.^8^ A mortalidade geral foi de 8,2%, embora a cirurgia valvar múltipla tivesse uma mortalidade operatória de 13%. As complicações pós-operatórias estavam presentes em mais da metade dos pacientes, sendo as mais comuns a ventilação prolongada em mais de um quarto.

No escore STS-IE, os números variam de 0 a 110 pontos e, de acordo com esse modelo, um paciente com 35 pontos teria um risco operatório de pelo menos 10% de mortalidade.^8^ Embora apenas pacientes com EI tenham sido estudados, esse foi um registro voluntário exclusivamente de hospitais americanos. Características importantes da EI, como microbiologia, discriminação entre válvulas nativas e próteses e presença de complicações intracardíacas (abscesso, fístula) não foram analisadas. Surpreendentemente, 43% dos pacientes foram operados de maneira “eletiva”, o que é um cenário diferente das outras séries.

Embora não sejam específicos para endocardite, o Euroscore e o Euroscore II levam em consideração a endocardite ativa como uma variável importante associada à mortalidade operatória (ver Tabela 1 ). É importante ressaltar que foram criados vários escores mais específicos para a endocardite, incluindo variáveis com peso significativo em relação à gravidade dessa condição,^8 - 13^ mostradas na tabela 1 do artigo de Pivatto Jr F et al.^1^ As características específicas da EI são: EI de válvula protética, grande destruição intracardíaca, *Staphylococcus spp.* , patógeno isolado na cultura de amostras de sangue (ou seja, hemoculturas positivas), presença de abscesso, complicações perivalvares, microrganismo virulento; além destes, há bloqueio atrioventricular e microrganismos Gram-negativos não-HACEK (os últimos 2 para o modelo INC-Rio^4^) e envolvimento perivalvar (por exemplo, abscesso anular ou fístula aortocavitária).^13^ Quando agrupados, além do envolvimento da prótese, essencialmente o tipo de microrganismo e a destruição da válvula (bloqueio AV sinalizando abscesso perivalvar) são as características distintivas desses “escores de EI” (ver Tabela 2 ). Mostramos mais dados sobre os escores estudados por Pivatto Jr F et al.,^1^ na Tabela 3 e adicionamos a isso os escores INC-Rio^4^ e DeFeo et al. ^13^ A mortalidade e a AUC dos escores, em relação à população estudada, são mostradas ( Tabela 3 ). Vale ressaltar que a mortalidade foi variável nas diferentes séries e a mortalidade em pacientes operados com EI é pelo menos o dobro da observada em outras cirurgias cardíacas (observe as menores taxas de mortalidade para as populações estudadas no Euroscore I e II). O presente estudo não propõe um escore, e foi adicionado à tabela para mostrar a mortalidade em sua série. Neste estudo^1^, a melhor taxa de mortalidade O/E foi alcançada pelo escore PALSUSE, seguido pelo EuroSCORE logístico, que demonstrou o maior poder discriminatório e foi significativamente superior ao EuroSCORE II, STS-IE, PALSUSE, AEPEI, e RISK-E.

**Tabela 2:** – Variáveis incluídas em escores prognósticos para mortalidade em cirurgia cardíaca em pacientes com endocardite infecciosa submetidos a troca valvar

Variáveis	PALSUSE2014	AEPEI 2017	INC-Rio 2016	EndoSCORE 2017	RISK-E 2017
Endocardite de valva protética					
Idade					
Grande destruição intracardíaca*					
*Staphylococcus* spp.					
Cirurgia urgente					
Sexo (feminino)					
EuroScore ≥10					
IMC > 27Kg/m^2^					
Estado pré-operatório crítico					
ClearCreat < 50mL/min					
Classe IV NYHA					
PSAP > 55mmHg					
DPOC					
Cr ≥ 2mg/dL					
FEVE					
Número de válvulas/próteses tratadas					
Microrganismo patogênico isolado em hemoculturas					
Presença de abscesso					
Insuficiência renal aguda					
***Choque cardiogênico***					
Complicações perivalvares^‡^					
Choque séptico					
Trombocitopenia^§^					
Microrganismo virulento^//^					
BAV					
DMID					
GN não-HACEK					
Uso de Inotrópicos					

*Abscessos ou outros achados de ecocardiografia sugeriram que a infecção foi invasiva (comunicação entre câmaras, dissecção de parede ou grande deiscência valvular). ‡ Abscesso, pseudoaneurisma, fistula ou deiscência protética; ^§^ < 150.000 plaquetas/mm^3^. ^//^Staphylococcus aureus ou fungos.

*IMC: índice de massa corporal; ClearCreat: clearance de creatinina (taxa de filtração glomerular estimada); NYHA: New York Heart Association; PSAP: pressão sistólica de artéria pulmonar; DPOC: doença pulmonar obstrutiva crônica; Cr: creatinina sérica; FEVE: fração de ejeção do ventrículo esquerdo; BAV: bloqueio atrioventricular; DMID: diabetes mellitus insulinodependente; GN: Gram Negativo; HACEK: Haemophilus spp, Aggregatibacter spp (anteriormente Actinobacillus), Cardiobacterium hominis, Eikenella corrodens, Kingella kingae.*

**Tabela 3:** – Áreas sob a curva (AUC) dos escores de risco propostos para avaliação de mortalidade em cirurgia cardíaca para endocardite infecciosa

ESCORE	AUC	Mortalidade pós- operatória intra-hospitalar	Avaliou EI separadamente?	N estudado	País	Autor, ano
Logistic EuroSCORE	0,79	4,7%	não	14.799	Países europeus*	Nashef 1999
EuroSCORE II	0,81	3,9%	não	22.381	Países europeus**	Nashef 2012
STS-IE	0,76	8,2%	sim	13.617	EUA	Gaca 2011
“De Feo”***	0,88	9,1%	sim	440	Itália	De Feo 2012
PALSUSE	0,68	24,3%	sim	437	Espanha	Martínez-Sellés 2014
INC-Rio	0,97	17,5%	sim	154	Brasil	Martins 2016
RISK-E	0,82	28,6%	sim	671	França e Espanha	Olmos 2017
AEPEI	0,78	15,5%	sim	361	França e Itália	Gatti 2017
EndoSCORE	0,85	11%	sim	2.715	Itália	Di Mauro 2017
Não foi criado	Não avaliada	29%	sim	107	Brasil	Pivatto Jr F, 2020

AUC: área sob a curva (area under the curve); EI: endocardite infecciosa. *Não está discriminado que países participaram. **A maior parte dos centros de investigação eram da França, Itália e Espanha; países da América do Sul e do Norte, Ásia e África tiveram participação pequena. EUA: Estados Unidos da América. *** Somente pacientes com EI de válvula nativa foram estudados; nenhum nome foi dado ao escore.

Em conclusão, vários grupos estão em busca de um escore adequado para prever a mortalidade em pacientes operados por EI. O Euroscore I e II, amplamente utilizados, e o STS-IE, foram estudados comparativamente aos novos escores propostos, alguns dos quais (por exemplo, PALSUSE) incluíram partes do Euroscore. No Brasil, apenas 2 estudos (o presente, com 107 pacientes, e o de Martins et al.,^4^ com 154) abordaram o desempenho dos escores na EI, ambos com pequeno número. No primeiro, os autores concluíram que, apesar da disponibilidade de escores específicos, o EuroSCORE logístico era o melhor para prever a mortalidade em sua coorte e nenhum escore foi proposto; no segundo, os escores de EI mencionados não foram avaliados (a maioria deles publicada após 2016), mas a sensibilidade e especificidade do Euroscore I foram de 81,5% e 63%; para Euroscore II, 29,6% e 97,6%, e para o STS-IE 7,4% e 98,4%, respectivamente. Os valores da AUC foram de 0,86 (Euroscore I), 0,90 (Euroscore II) e 0,85 (STS-IE). Na análise multivariada, as variáveis consideradas estatisticamente significantes para óbito foram bloqueio AV, choque cardiogênico, diabetes mellitus insulinodependente, microrganismos Gram-negativos não-HACEK e uso de inotrópicos. Estes foram incluídos em um modelo, o INC-Rio,^4^ com sensibilidade calculada de 88,9% e especificidade de 91,8%; a AUC foi de 0,97. Casalino et al.^14^ estudaram todos os tipos de cirurgia valvar em 440 pacientes, nos quais a taxa de mortalidade foi de 16,0% (6,0% em cirurgia eletiva e 34,0% em cirurgia de emergência / urgência) e constataram que a AUC foi de 0,76 para o EuroSCORE aditivo e logístico e 0,81 para o EuroSCORE II. Eles concluíram que os modelos de EuroSCORE tinham boa capacidade discriminatória, embora a calibração estivesse comprometida devido à subestimação da mortalidade.

Acreditamos que um estudo multinacional no Brasil seria de suma importância, com maior número de pacientes, para propor e validar um escore, uma vez que os pacientes com EI em nosso país diferem drasticamente daqueles dos países da América do Norte ou da Europa, principalmente devido à alta proporção de valvopatia reumática, EI causada por estreptococos do grupo *viridans* , maior demora no diagnóstico e idade mais jovem.
