# Supplementary material for: Escores Prognósticos de Mortalidade na Cirurgia Cardíaca para Endocardite Infecciosa
Source: Arq Bras Cardiol. 2020 Apr 6;114(3):525–9. [Article in Portuguese] doi: 10.36660/abc.20200070 (PMC7792738; doi:10.36660/abc.20200070)
Supplement: Translation

# Translation: Prognostic Scores for Mortality in Cardiac Surgery for Infective Endocarditis

**Authors:** Alexandre Bahia Barreiras Martins, Cristiane da Cruz Lamas

**Affiliations:** 1 Brazil Unidade Cardiointensiva - Clínica São Vicente da Gávea, Rio de Janeiro, RJ - Brazil; 2 Brazil Coordenação de Ensino e Pesquisa - Instituto Nacional de Cardiologia, Rio de Janeiro, RJ - Brazil; 3 Brazil Escola de Ciências da Saúde - Universidade do Grande Rio (Unigranrio), Rio de Janeiro, RJ - Brazil; 4 Brazil Centro Hospitalar - Instituto Nacional de Infectologia Evandro Chagas - Fiocruz, Rio de Janeiro, RJ – Brazil

**Keywords:** Endocarditis/surgery, Hospital Mortality, Cardiac Surgery/mortalidade, Prognosis, Scores

The article by Pivatto F Jr et al.^1^allows us to discuss the important issue of prognostic scores in patients who have cardiac surgery for infective endocarditis (IE).^1^The management of left- sided IE often involves surgery during the index admission, and the main challenge is to rapidly and correctly identify patients at high risk and to transfer them to institutions with a surgical team with expertise in endocarditis surgery.

Prognostic scores are important for several reasons: a reasonable estimate of the risk of death is important in clinical decision-making regarding surgical indication; the estimate is necessary to inform patients and their families of the surgical risk; risk stratification permits a fair comparison of cardiac surgery results, so that surgeons and hospitals treating high-risk patients will not appear to have worse results than others.^2^For operative mortality to remain a valid measure of quality of care, it must be related to the risk profile of the patients receiving surgery.^2^

Euroscore I, published in 1999, evaluated 19,030 patients submitted to cardiac surgery in 8 countries in Europe, studying 97 risk factors for death, and among those, the ones that significantly affected surgical prognosis were selected.^2^These variables are presented in Table 1 . In this study, only 30% were submitted to valve surgery, and the number of individuals who had endocarditis is not mentioned.^2^

**Table 1:** – Variables included in prognostic scores for cardiac surgery (Euroscore I and II and STS-IE)

Variables	EuroScore I	EuroScore II	STS-IE Score
Age			
Gender			
Weight			
Height			
Body Mass Index			
Diabetes Mellitus			
Chronic Pulmonary Disease			
Extracardiac arteriopathy			
Peripheral Arterial Disease			
Neurological Dysfunction			
Low Mobility			
Previous cardiac surgery			
Number of previous surgeries			
Previous valvar surgery			
Renal failure under conservative treatment			
Renal Failure under Hemodialysis			
Serum Creatinine / Creatinine Clearance			
Arrythmia			
Systemic Arterial Hypertension			
***Active infective endocarditis***			
Immunosuppressive therapy			
Recent myocardial infarction			
Cardiogenic shock			
Inotropic use			
Intra-aortic balloon			
NYHA (New York Heart Association) Classification			
Non-coronary surgery			
Unstable Angina (CCS IV)			
Preoperative Critical State			
Left Ventricular Ejection Fraction			
Pulmonary Arterial Hypertension			
Resuscitation			
Urgency Procedure			
Intervention weight:			
Single non-coronary procedure			
2 Procedures			
3 Procedures			
Septal rupture after myocardial infarction				

Euroscore II, published in 2012,^3^had the goal of updating the first model by evaluating 22,381 patients from 43 countries in the world, including sites outside Europe, so as to create a more reliable score, incorporating new variables and adjusting others ( Table 1 ). At this time, it was already known that the Euroscore^2^superestimated the surgical risk as technical progress in cardiac surgery along the previous decade had been made, with a mortality decrease adjusted by risk. Improvements to Euroscore were: creatinine clearance as a better measure of renal function than serum creatinine values; unstable angina defined by the use of intravenous nitrates was outdated; weight of intervention was not properly assessed in the previous model (for example, aortic valve replacement with or without concurrent coronary artery bypass grafting had the same weight) and some continuous variables, such as number of previous cardiac surgeries and pulmonary artery systolic pressures were treated as a dichotomic variable.^3^

The receiving operator curve (ROC) of the scores showed an area under the curve (AUC) of 0.78 for the logistic and additive Euroscore and of 0.80 for Euroscore II. A criticism to the model is, that although non-European countries were included, the vast majority of patients were from Spain, France, Italy and the United Kingdom, who contributed with 19, 16, 15 and 12 sites respectively.^3^As for Latin America, Brazil contributed with data from 4 centers, Argentina 1 and Uruguay 1. Also, the model did not analyze valve surgery separately. In fact, only 2.2% of patients (497 in absolute numbers) with active IE had been included.^4^A limitation outlined in the study was that all centers participated voluntarily, what introduces selection bias to the data.^3^

Patients with IE must be thoroughly assessed. If we consider the usual profile of a patient with IE who is operated at Instituto Nacional de Cardiologia, for example, he or she will have a serum creatinine above normal, scoring 2 points; active disease (under antibiotic treatment for IE at the time of surgery), scoring 3 points, and at least moderate left ventricular dysfunction, scoring 1, that is, with a total Euroscore of 6 and anticipated mortality of over 11%. Not infrequently, this patient previously had cardiac surgery (as over a third have rheumatic valvopathy and about 10% previously had IE), which adds 3 points to the total score.^4 - 6^Therefore, Euroscore I does not discriminate well this subset of patients, as most will probably fall into the 6+ score. Patrat-Delon et al.,^7^studying 149 patients operated for IE in France, between 2002 and 2013, of which in-hospital mortality was 21%, came to a similar conclusion regarding EuroSCORE II: it underestimated mortality in patients with predicted mortality over 10%.^7^

The Society of Thoracic Surgeons–Infective Endocarditis (STS-IE) score, published in 2011,^8^has its variables shown schematically in Table 1 . In the subset of North American patients with IE studied in its development, of the 13,617 patients, only over half had active endocarditis at the time of surgery.^8^Overall mortality was 8.2%, although multiple valve surgery had an operative mortality of 13%. Postoperative complications were present in more than half the patients, most common of which were prolonged ventilation in over a quarter.

In the STS-IE score, numbers vary from 0-110 points and, according to this model, a patient with 35 points would have an operative risk of at least 10% mortality.^8^Although only patients with IE were studied, this was a voluntary registry of American hospitals only. Important features of IE, such as microbiology, the discrimination between native and prosthetic valves and the presence of intracardiac complications (abscess, fistula) were not analyzed. Surprisingly, 43% of the patients were operated on “electively”, which is a different scenario from other series.

Although not specific for endocarditis, Euroscore and Euroscore II take into account active endocarditis as an important variable associated with operative mortality (see Table 1 ). Importantly, several scores have been created, which are more specific to endocarditis, involving variables that carry a significant weight regarding severity of this condition,^8 - 13^shown in table 1 of the article by Pivatto Jr F et al.^1^Features specific to IE are prosthetic valve IE, large intracardiac destruction, *Staphylococcus* spp., pathogen isolated from a blood specimen culture (i.e., positive blood cultures), presence of abscess, perivalvar complications, virulent microorganism; besides these, there is atrioventricular block and non-HACEK Gram negatives (the last 2 for INC-Rio model^4^) and perivalvular involvement (ex. annular abscess or aortocavitary fistula).^13^When grouped, in addition to prosthesis involvement, essentially type of microorganism and valve destruction (AV block signaling perivalvular abscess) are the distinctive features in these “IE scores” ( see Table 2 ). We have shown more data on the scores studied by Pivatto Jr F et al.^1^in table 3 , and we have added to this the INC-Rio^4^and the DeFeo scores.^13^Mortality and AUC of the scores, relative to their studied population, are shown ( Table 3 ). It is noteworthy that mortality was variable in the different series, and mortality in patients operated with IE was at least double that seen in other types of cardiac surgery (note the lower mortality rates for the populations studied in Euroscore I and II). The present study does not propose a score, and it was added to the table so as to show mortality in their series. In this study^1^, the best O/E mortality ratio was achieved by the PALSUSE score, followed by the logistic EuroSCORE, which had the highest discriminatory power and was significantly superior to EuroSCORE II, STS-IE, PALSUSE, AEPEI and RISK-E.

**Table 2:** – Variables included in prognostic scores for cardiac surgery mortality in patients with infective endocarditis undergoing valve replacement

Variables	PALSUSE2014	AEPEI 2017	INC-Rio 2016	EndoSCORE 2017	RISK-E 2017
Prosthetic valve endocarditis					
Age					
Large intracardiac destruction*					
*Staphylococcus spp.*					
Urgent surgery					
Gender (female)					
EuroScore ≥10					
BMI > 27Kg/m^2^					
Critical preoperative state					
ClearCreat < 50mL/min					
Class IV NYHA					
PASP > 55mmHg					
COPD					
Cr ≥ 2mg/dL					
LVEF					
Number of valves / prostheses treated					
Pathogenic microorganism isolated in blood cultures					
Presence of abscess					
Acute renal failure					
Cardiogenic shock					
Perivalvar complications^‡^					
Septic shock					
Thrombocytopenia^§^					
Virulent microorganism^//^					
Atrioventricular block					
IDDM					
Non-HACEK Gram negatives					
Inotropic use					

*Abscesses or other echocardiographic findings suggested that the infection was invasive (inter-chamber communication, wall dissection or major valve dehiscence).

*^‡^Abscess, pseudoaneurysm, prosthetic fistula or dehiscence;^§^< 150,000 platelets/mm^3^.*

*^//^Staphylococcus aureus or fungi.*

*BMI: body mass index; ClearCreat = creatinine clearance (estimated glomerular filtration rate); NYHA: New York Heart Association; PASP = pulmonary artery systolic pressure; COPD = chronic obstructive pulmonary disease; Cr = serum creatinine; LVEF = left ventricular ejection fraction; AVB: atrioventricular block; IDDM = insulin-dependent diabetes mellitus; GN: Gram-Negative; HACEK = Haemophilus spp, Aggregatibacter spp (formerly Actinobacillus), Cardiobacterium hominis, Eikenella corrodens, Kingella kingae.*

**Table 3:** – Areas under the curve (AUC) of the proposed risk scores for assessing mortality in cardiac surgery for infective endocarditis

SCORE	AUC	Postoperative intrahospital mortality	Evaluated IE separately?	N. studied	Country	Author, year
Logistic EuroSCORE	0.79	4.7%	no	14,799	European countries*	Nashef 1999
EuroSCORE II	0.81	3.9%	no	22,381	European countries**	Nashef 2012
STS-IE	0.76	8.2%	yes	13,617	USA	Gaca 2011
“De Feo”***	0.88	9.1%	yes	440	Italy	De Feo 2012
PALSUSE	0.68	24.3%	yes	437	Spain	Martínez-Sellés 2014
INC-Rio	0.97	17.5%	yes	154	Brazil	Martins 2016
RISK-E	0.82	28.6%	yes	671	France and Spain	Olmos 2017
AEPEI	0.78	15.5%	yes	361	France and Italy	Gatti 2017
EndoSCORE	0.85	11%	yes	2,715	Italy	Di Mauro 2017
Not created	Not assessed	29%	yes	107	Brazil	Pivatto Jr F, 2020

AUC: area under the curve; IE: infective endocarditis. *Participating countries were not discriminated. **Most of the research centers were located in France, Italy and Spain; countries in South and North America, Asia and Africa had a small participation. USA: United States of América. ***Only patients with native valve IE were studied; no name was given to the score.

In conclusion, several groups are in search of an adequate score to predict mortality in patients operated for IE. The widely used Euroscore I and II, and the STS-IE have been studied comparatively to the new proposed scores, some of which (for ex., PALSUSE) have included parts of Euroscore to them. In Brazil, only 2 studies (the present one, with 107 patients, and the one by Martins et al.^4^with 154) have addressed the performance of scores in IE, both with small numbers. In the first, the authors concluded that, despite the availability of specific scores, the logistic EuroSCORE was the best to predict mortality in their cohort and no score was proposed; in the second, the mentioned IE scores were not evaluated (most of them published after 2016), but the sensitivity and specificity of Euroscore I was 81.5% and 63%; for Euroscore II , 29.6% and 97.6%, and for STS-IE 7.4% and 98.4%, respectively. AUC values were 0.86 (Euroscore I), 0.90 (Euroscore II) and 0.85 (STS-IE). In the multivariate analysis, the variables found to be statistically significant for death were AV block, cardiogenic shock, insulin-dependent diabetes mellitus, non-HACEK Gram negative microorganisms and inotropic use. These were included in a model, INC-Rio^4^with a calculated sensitivity of 88.9% and specificity of 91.8%; AUC was 0.97. Casalino et al.^14^have studied all-type valvular surgery in 440 patients, in which mortality rate was 16.0% (6.0% in elective surgery and 34.0% in emergency/urgency surgery), and found the AUC was 0.76 for additive and logistic EuroSCORE and 0.81 for EuroSCORE II. They concluded that the EuroSCORE models showed good discriminatory capacity, although calibration was compromised due to mortality underestimation.

We believe a multinational study in Brazil would be of paramount importance, with a greater number of patients, to propose and validate a score, since patients with IE in our country dramatically differ from those in North American or European countries, especially due to the high proportion of rheumatic valvopathy, group *viridans* streptococcal IE, longer delay time to diagnosis, and younger age.
